# Effects of global warming on Mediterranean coral forests

**DOI:** 10.1038/s41598-021-00162-4

**Published:** 2021-10-19

**Authors:** Giovanni Chimienti, Diana De Padova, Maria Adamo, Michele Mossa, Antonella Bottalico, Anna Lisco, Nicola Ungaro, Francesco Mastrototaro

**Affiliations:** 1 Department of Biology and CoNISMa LRU, University of Bari Aldo Moro, Via Orabona 4, 70125 Bari, Italy; 2 CoNISMa, Rome, Italy; 3 DICATECh, Polytechnic University of Bari, Bari, Italy; 4 Institute of Atmospheric Pollution Research (IIA), National Research Council (CNR), Bari, Italy; 5 Apulian Regional Agency for the Environmental Prevention and Protection, Bari, Italy

**Keywords:** Climate-change ecology, Conservation biology, Marine biology, Environmental health

## Abstract

The effects of global warming have been addressed on coral reefs in tropical areas, while it is still unclear how coral forests are reacting, particularly at temperate latitudes. Here we show how mesophotic coral forests are affected by global warming in the Mediterranean Sea. We highlight how the current warming trend is causing the lowering of the thermocline and it is enhancing mucilaginous blooms. These stressors are facilitating a massive macroalgal epibiosis on living corals, here reported for the first time from different areas in the Western and Central Mediterranean Sea. We provide a focus of this phenomenon at Tremiti Islands Marine Protected Area (Adriatic Sea), were the density of the endemic red gorgonian *Paramuricea clavata* decreased of up to 47% in 5 years, while up to the 96% of the living corals showed signs of stress and macroalgal epibiosis. Only populations deeper than 60 m depth were not touched by this emerging phenomenon. Spot observations performed at Tuscan Archipelago and Tavolara Marine Protected Area (Tyrrhenian Sea) suggest that this this combination of stressors is likely widespread at basin scale.

## Introduction

Rising sea surface temperatures related to global warming are increasingly affecting marine habitats and people who depend on them^1,2^. This is particularly evident on coral reefs, due to the great ecological importance of this habitat and the strong visual phenomenon of coral bleaching that often precedes coral mortality events^3–6^. Reef-forming corals are less predominant in temperate coastal ecosystems, where arborescent corals, such as those belonging to orders Alcyonacea and Antipatharia, often play a key structural role. These corals can create monospecific or mixed coral forests that represent recovery, feeding and nursery areas for many associated species^7–9^. The Food and Agriculture Organization (FAO) identified coral forests as Vulnerable Marine Ecosystems (VME)^10^, although these habitats have been scantly mapped and monitored thus far.

The Mediterranean Sea is a semi-enclosed basin that hosts more than 7% of world’s marine biodiversity, including many endemic species^11^, but where global warming is causing drastic changes on many compartments, with considerable impacts^12–15^. Mediterranean species have generally a cold affinity and are particularly sensitive to increasing temperatures, representing a wake-up call for the effects of climate changes at temperate latitudes. The first evidences of coral Mass Mortality Events (MMEs) due to sea surface temperature rise in Mediterranean coastal ecosystems date back to ~ 40 years ago^16,17^. Since then, several other MMEs have been reported (e.g.^18–23^), many of which have been associated with marine heat waves^24–26^, which are becoming more frequent globally^2,27^.

The red gorgonian or red sea fan *Paramuricea clavata* (Risso, 1826) is a long‐lived, slow‐growing species characterized by colonies irregularly branched, purple/yellow in colour, that can exceed 1.5 m in height and live for over a century^28,29^. This species is characteristic of the mesophotic zone of the Mediterranean Sea, where it forms coral forests of great ecological importance, supporting high species diversity and promoting bioconstruction processes^30^. It is endemic of the basin and classified as “vulnerable” according to the International Union for the Conservation of Nature^31^. Here we describe an extensive MME of *P. clavata* in the Adriatic Sea. We focused on Tremiti Islands Marine Protected Area (MPA) due to its luxuriant population of forest-forming corals such as gorgonians and antipatharians^9^, as well as to exclude the direct role of fishing pressures in the mortality event. Measures and modelling of water temperature in the last decade allowed to correlate the mortality event to the rising of water temperature at mesophotic depths, including the displacement in depth of the lower limit of the thermocline. Heatwaves and global warming^32^, together with massive mucilaginous aggregates and macroalgal overgrowth on living corals, represent a combination of stressors that are threatening coastal coral forests in an unprecedented way. The observation of similar processes in other areas of the Mediterranean suggests that this phenomenon may be happening at basin scale, affecting also other coral species.

## Results

### Mass mortality of *Paramuricea clavata* at Tremiti Archipelago

**Figure 1:**
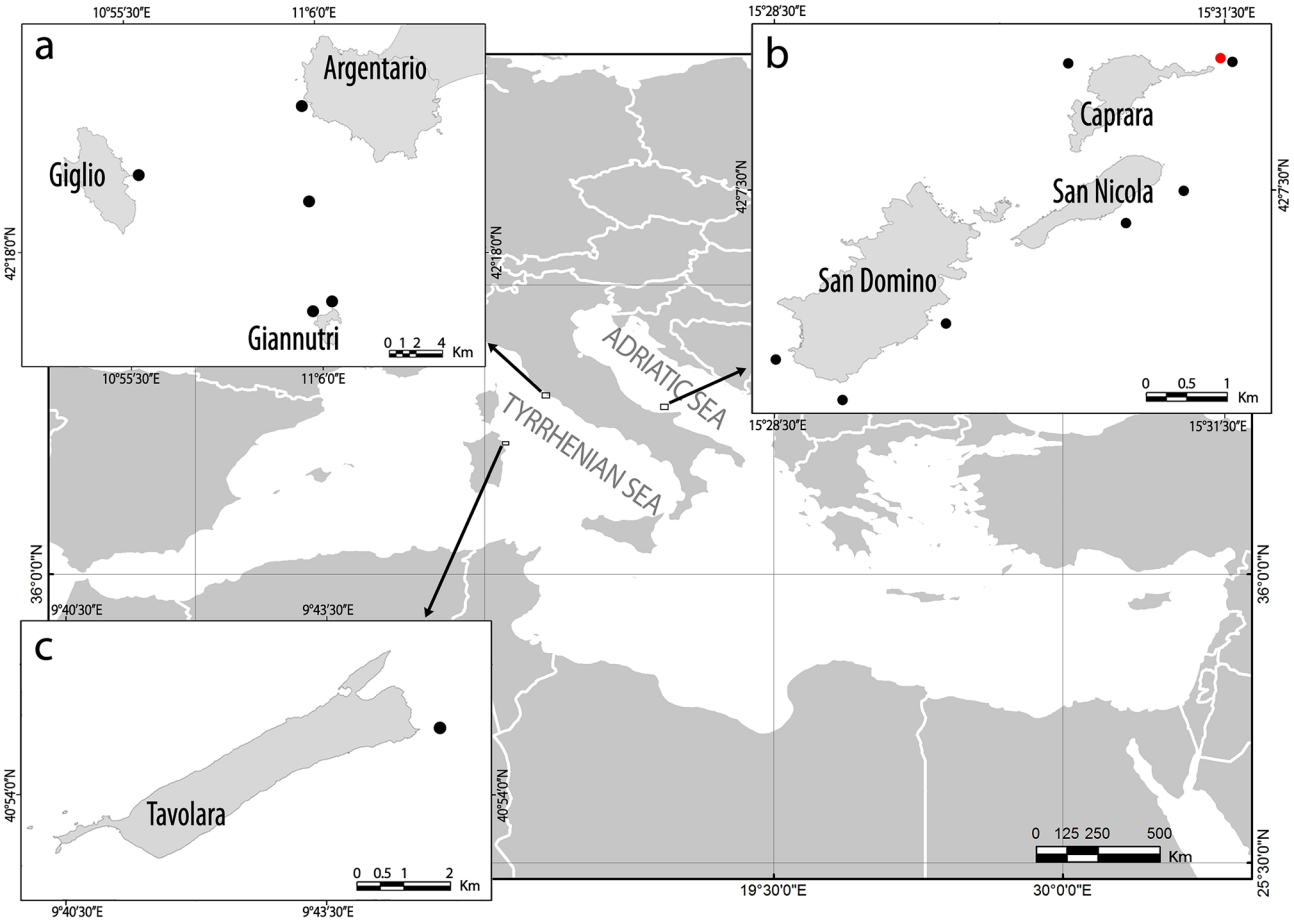
Map of the study areas, with details of (**a**) Tuscan Archipelago; (**b**) Tremiti Archipelago; (**c**) Tavolara Island. The study site of Punta Secca shoal is represented with a red dot, while the other unquantified occurrences of macroalgal epibiosis on gorgonians are represented with black dots. Maps have been created using ESRI ARCMAP 10.2 (https://support.esri.com/en/products/desktop/arcgis-desktop/arcmap/10-2-2).

**Figure 2:**
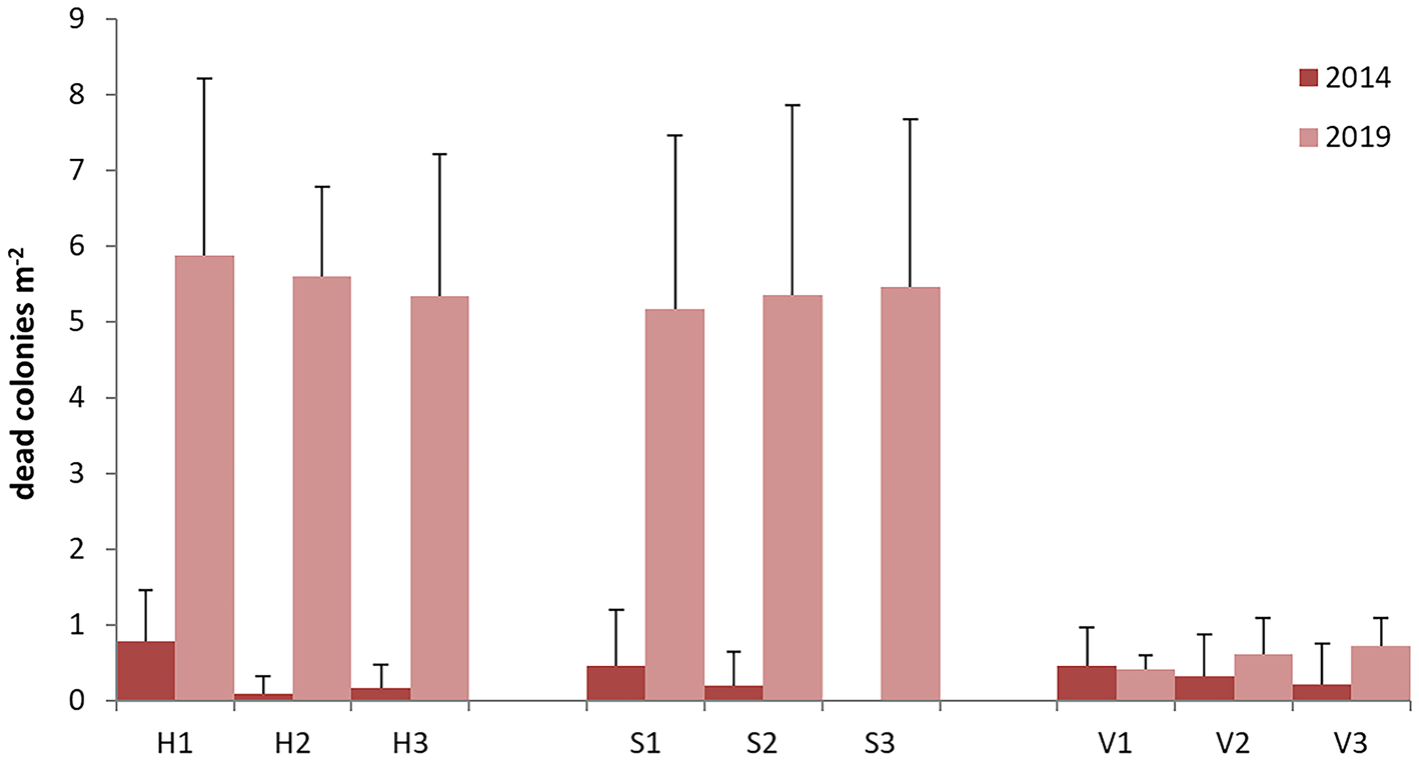
Mortality (mean ± standard error) of *Paramuricea clavata* during 2014 and 2019 at Tremiti Archipelago, with three replicates per area characterized by horizontal (H), sub-vertical (S) and vertical (V) substrate.

**Figure 3:**
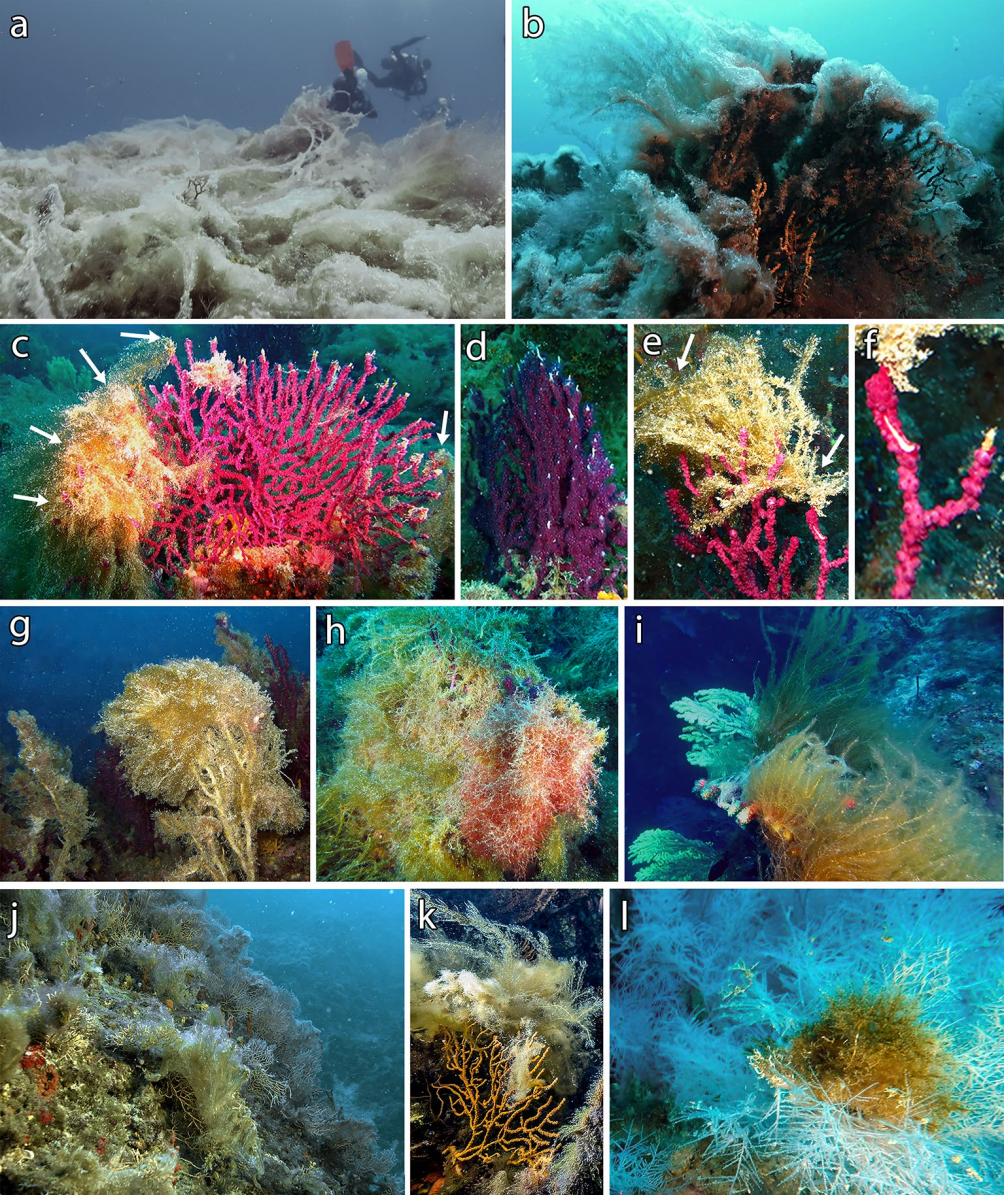
Effects of global warming on coral forests at Tremiti Islands. (**a**) Massive mucilaginous aggregates covering a forest of *Paramuricea clavata* (2017); (**b**) Colony of *P. clavata* with mucilage and *Sporochnus pedunculatus* (2018); (**c**–**e**) Colonies with extremities necrosis and *S. pedunculatus* epibiosis (arrows) (2019); (**f**) Detail of extremities lesion and necrosis on; (**g**) Dead colony covered by epibionts (2019); (**h**) Living colony completely covered by macroalgae (2019); (**i**) Living colonies on the top of a rocky wall, with *S. pedunculatus* growing on the side of the corals exposed to light (2019); (**j**) Forest of *Eunicella cavolini* affected by mucilage and *S. pedunculatus*, with (**k**) detail of one colony (2018); (**l**) Macroalgal epibionts on *Antipathella subpinnata* (2018).

Tremiti Islands MPA proved to be a hotspot for *P. clavata* presence in the Adriatic Sea, with shoals such as Punta Secca (Fig. 1) hosting a population of hundreds thousands colonies over 35,000 m^2^, from 35 to 70 m depth. The ca. 2,000 colonies of *P. clavata* monitored during five years (2014–2019) on this shoal, over an area of 200 m^2^, underwent a large MME. In particular, colonies were healthy in the first year of monitoring, with very low mortality ranging from 0.08 ± 0.24 to 0.77 ± 0.67 colonies m^−2^ (mean ± standard error) all over the area (Fig. 2). Afterwards, massive mucilaginous blooms occurred from 2015, being particularly severe and persistent in the summer of both 2017 and 2018. The gorgonians on the top and the slope of the shoal, from 32 to 40 m depth, were entirely covered with mucilage to such an extent that it was not possible to quantify colonies density and mortality using visual methods (Fig. 3a). Colonies deeper than 40 m and settled on vertical rocky bottoms were broadly not touched by this phenomenon that lasted, at least, from June to September during both years. From 2018, macroalgal skeins were observed colonizing living colonies of *P. clavata* for the first time. The brown alga *Sporochnus pedunculatus* (Hudson) C. Agardh (Fig. 3b) was the most common and became extremely abundant during 2019. Gorgonians exposed to *S. pedunculatus* epibiosis and overgrowth showed necrosis of the living tissues that became more susceptible to colonization by other epibionts.

**Figure 4:**
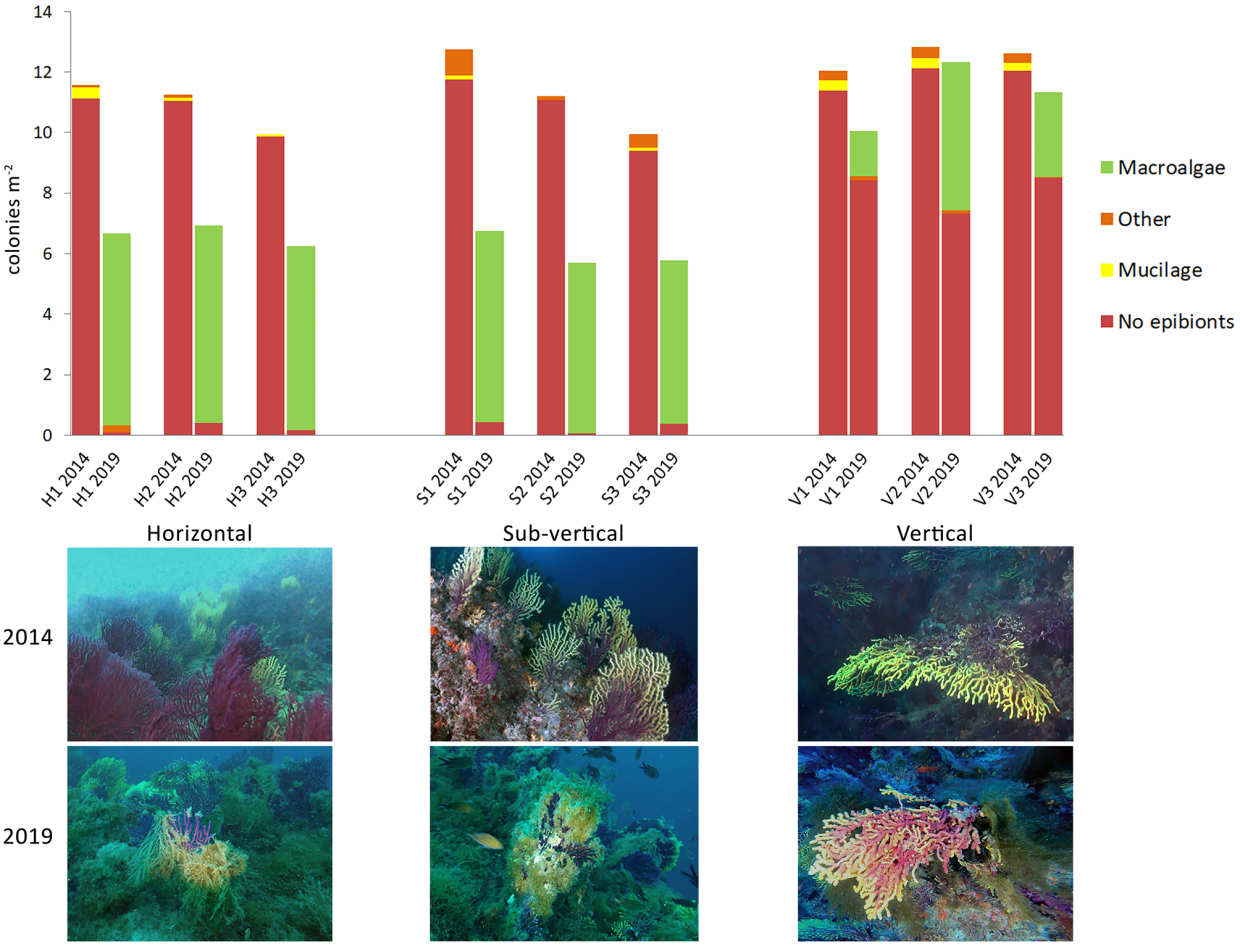
Colonies density of *Paramuricea clavata* on the three portions of the shoal, with three different inclinations of the seabed, surveyed during 2014 and 2019 (three study sites each). Densities of the colonies affected by mucilage and epibionts are provided, with representative photos of the same portions on the shoal during both 2014 and 2019 (below).

Density assessments were possible only in 2014 and 2019. The population of *P. clavata* settled on horizontal substrate (top of the shoal, 32–35 m depth) was highly affected by macroalgal epibiosis. From 2014 to 2019, 39.4% of colonies died (Table S1) and the majority of the living ones was characterized by the necrosis of the branch extremities during 2019 (Fig. 3c–f). All the dead colonies were completely covered by epibionts (Fig. 3g). The living colonies were massively colonized by macroalgae, mostly *S. pedunculatus*, that affected 95.5% of the monitored colonies (Figs. 3h, 4). A similar scenario was found on sub-vertical substrate (slope of the shoal, 35–38 m depth), where 47.2% of the colonies was dead and 95.6% of the living ones was covered by macroalgal skeins during 2019. On vertical substrate (shoal flank, 38–41 m depth), most of the colonies impacted were those at the upper portion of the wall (Fig. 3i), while those at lower layers were generally healthier. Only 9.9% of the monitored colonies was dead in five years, and macroalgae affected 27.3% of the living colonies during 2019. Replicates within each of the three substrates were homogeneous in both years. Colony densities resulted statistically different between 2014 and 2019 at all the three substrates considered (horizontal: *p* < 0.001; slope: *p* < 0.001; vertical: *p* < 0.05).

Other seven shoals monitored around the archipelago (Fig. 1) displayed a similar situation, although not quantified in detail. All *P. clavata* forests of the archipelago, as well as those of *Eunicella cavolini* (Koch, 1887), were massively covered with mucilage during 2017 and 2018, displaying macroalgal epibiosis during 2018–2019 (Fig. 3j–k) and consequent mortality. Coral forests between 40 and 60 depth were scarcely affected by macroalgal epibiosis, while those settled deeper than 60 m were not affected, regardless of the inclination of the substrate. One colony of the black coral *Antipathella subpinnata* (Ellis & Solander, 1786) at 58 m depth was the deepest observed record of macroalgae growing as epibiont on a coral (Fig. 3l).

### Macroalgal epibionts

**Figure 5:**
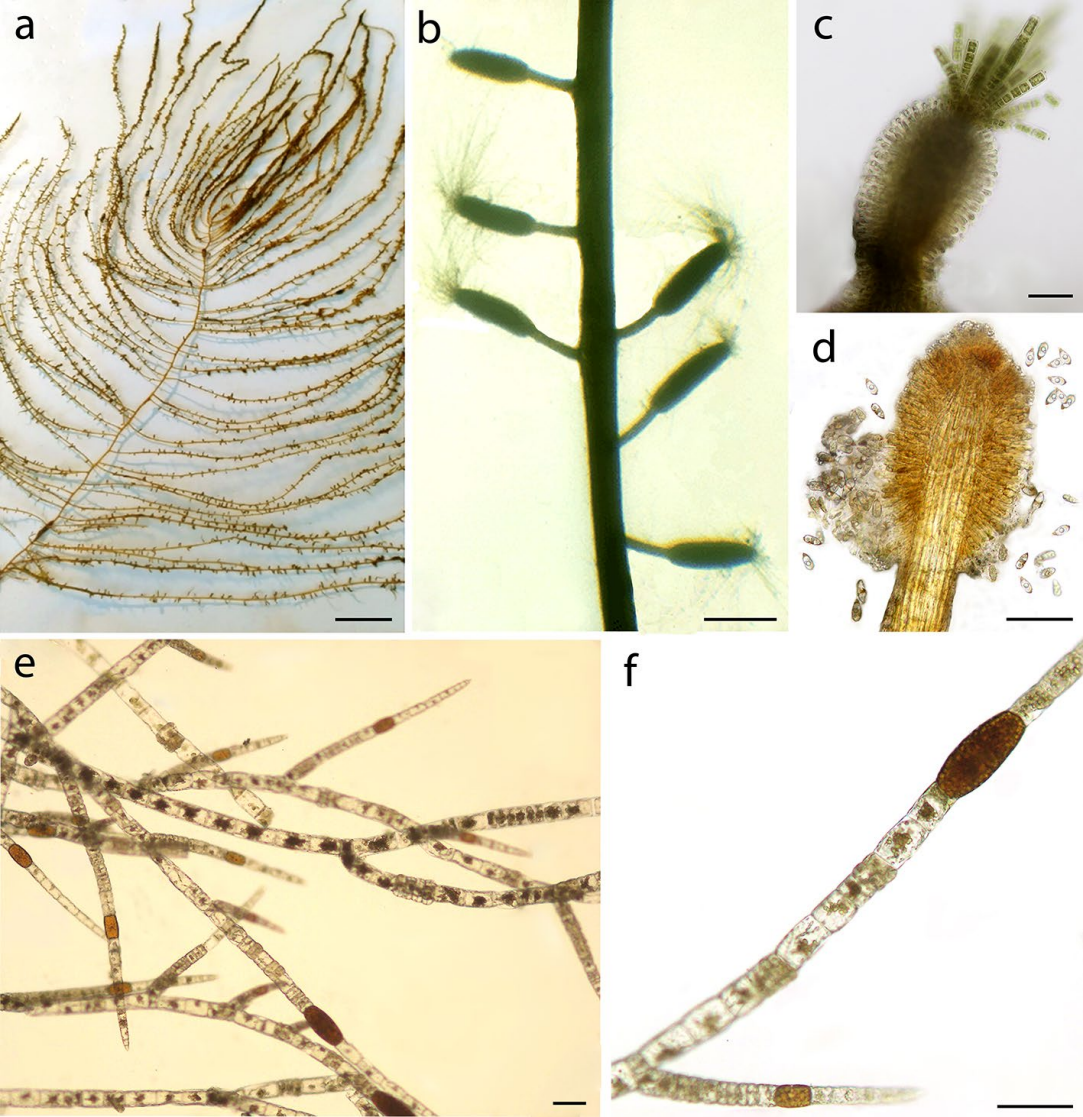
Details of the two most common macroalgae found on gorgonians. *Sporochnus pedunculatus*: (**a**) Habit; (**b**) Portion of thallus showing stalked receptacles; (**c**) Receptacle with a terminal tuft of assimilatory filaments; (**d**) Zoospores released from mature sporangia. *Pylaiella littoralis*: (**e**) Branching pattern; (**f**) Fertile filament showing intercalary plurilocular sporangia. Scale bars: (**a**) 5 cm; (**b**) 1 mm; (**c**–**f**) 100 µm.

Algal skeins collected on living portions of *P. clavata* were mainly composed of *S. pedunculatus* (53%) and *Pylaiella littoralis* (Linnaeus) Kjellman (16%). Thalli of *S. pedunculatus* were up to 50 cm long, greenish or brownish in colour, with a single terete main axis (1–2 mm in diameter), tapering towards the apex and giving rise to one order of indeterminate laterals, each characterized by many determinate branchlets (Fig. 5a). Primary lateral branches were numerous, regularly spaced, thinner than the main axis, irregularly alternate and radially arranged. All branches were terminated by short tufts of assimilatory filaments. Receptacles were ovoid to clavate, 0.5–1 mm long, at first sessile, then borne on stalks shorter than 1 mm (Fig. 5b). A tuft of assimilatory filaments, about 20–30 µm wide, arose from the apex of each receptacle (Fig. 5c). Sori consisted of paraphyses terminated by an inflated subspherical cell and laterally bearing unilocular sporangia (8–10 × 20–25 µm), releasing at maturity many zoospores (Fig. 5d). Thalli of *P. littoralis* were filamentous and formed tufts up to 2.5 cm in height. Filaments were uniseriate, irregularly branched, with branches unilaterally arranged in the upper portions (Fig. 5e). Unilocular and plurilocular sporangia (100–250 × 20–40 µm) (Fig. 5f) were intercalary. Other algae and bryozoans (mainly belonging to the genus *Savignyella*) were growing as epibionts on the two main algal species (Table 1).

**Table 1:** List of algal epibionts occurring in skeins covering the living *Paramuricea clavata* colonies or settled on the necrotic extremities of the coral branches.

Taxa	Skeins	Necrotic extremities
**Rhodophyta**		
*Acrosorium ciliolatum* (Harvey) Kylin		x
*Antithamnion cruciatum* (C. Agardh) Nägeli	x	x
*Antithamnion tenuissimum* (Hauck) Schiffner		x
*Antithamnionella elegans* (Berthold) J.H. Price & D.M. John *	x	x
*Antithamnionella spirographidis* (Schiffner) E.M. Wollaston *	x	x
*Apoglossum ruscifolium* (Turner) J. Agardh	x	x
*Asparagopsis armata* Harvey (only as *Falkenbergia* phase) *		x
*Balliella cladoderma* (Zanardini) Athanasiadis		x
*Centroceras clavulatum* (C. Agardh) Montagne		x
*Ceramium bisporum* D.L. Ballantine *	x	x
*Ceramium cimbricum* H.E. Petersen		x
*Ceramium cimbricum* f. *flaccidum* (H.E. Petersen) G. Furnari & Serio		x
*Ceramium codii* (H. Richards) Mazoyer		x
*Ceramium comptum* Børgesen		x
*Ceramium giacconei* Cormaci & Furnari		x
*Crouania attenuata* (C. Agardh) J. Agardh		x
*Dasysiphonia* sp.	x	x
*Griffithsia opuntioides* J. Agardh		x
*Hypoglossum hypoglossoides* (Stackhouse) Collins & Hervey		x
*Laurencia obtusa* (Hudson) J.V. Lamouroux		x
*Melobesia membranacea* (Esper) J.V. Lamouroux		x
*Pneophyllum fragile* Kützing		x
*Polysiphonia atlantica* Kapraun & J.N. Norris *	x	x
*Seirospora apiculata* (Meneghini) G. Feldmann-Mazoyer		x
*Womersleyella setacea* (Hollenberg) R.E. Norris *	x	x
*Wurdemannia miniata* (Sprengel) Feldmann & Hamel		x
**Chlorophyta**		
*Pedobesia simplex* (Meneghini *ex* Kützing) M.J. Wynne & F. Leliaert		x
*Valonia utricularis* (Roth) C. Agardh		x
**Ochrophyta-Phaeophyceae**		
*Dictyota dichotoma* (Hudson) J.V. Lamouroux	x	x
*Pylaiella littoralis* (Linnaeus) Kjellman *	x	x
*Sphacelaria cirrosa* (Roth) C. Agardh		x
*Sphacelaria fusca* (Hudson) S.F. Gray		x
*Sporochnus pedunculatus* (Hudson) C. Agardh	x	x

Non-indigenous species are marked with an asterisk.

A total of 33 taxa of macroalgae was identified on the apical fragments of *P. clavata*: 26 Rhodophyta, 2 Chlorophyta and 5 Ochrophyta-Phaephyceae, including seven non-indigenous species (Table 1). Besides *S. pedunculatus*, the erect algae *P. littoralis* and *Dasysiphonia* sp., as well as the turf algae *Polysiphonia atlantica* Kapraun & J.N. Norris and *Womersleyella setacea* (Hollenberg) R.E. Norris were the most abundant. These taxa showed the tendency of colonizing *P. clavata* from the necrotic extremities of the branches, then gradually covering the whole exposed axis of the gorgonian and its living tissues.

### Measured and modelled water temperature

**Figure 6:**
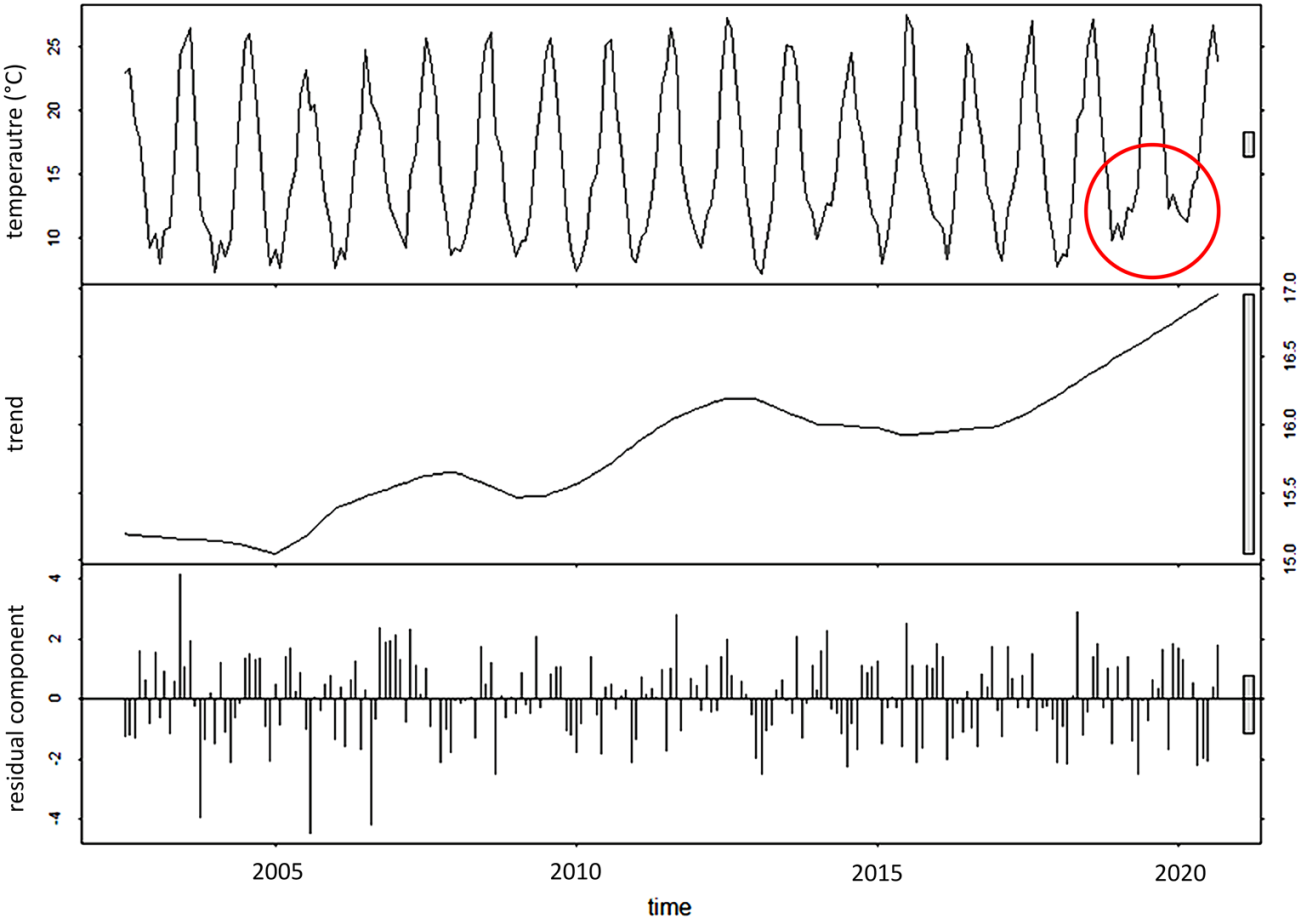
MODIS Aqua sea surface temperature at Tremiti archipelago (Adriatic Sea), between 2002 and 2020, with indication of the trend and the residual component. The red circle shows high surface temperatures during winter 2019 and 2020.

The time series of MODIS Aqua sea surface temperature (SST) at Tremiti Islands showed a general increasing of the SST in the last 20 years, that became more marked from 2016 due to higher winter/colder SST values (Fig. 6). Minimum values of water temperatures contributed to this trend, with 2020 showing the highest winter SST in the last twenty years. Residual component did not show any particular pattern, thus representing the irregular variation of SST measurements due to random disturbance (Fig. 6).

**Figure 7:**
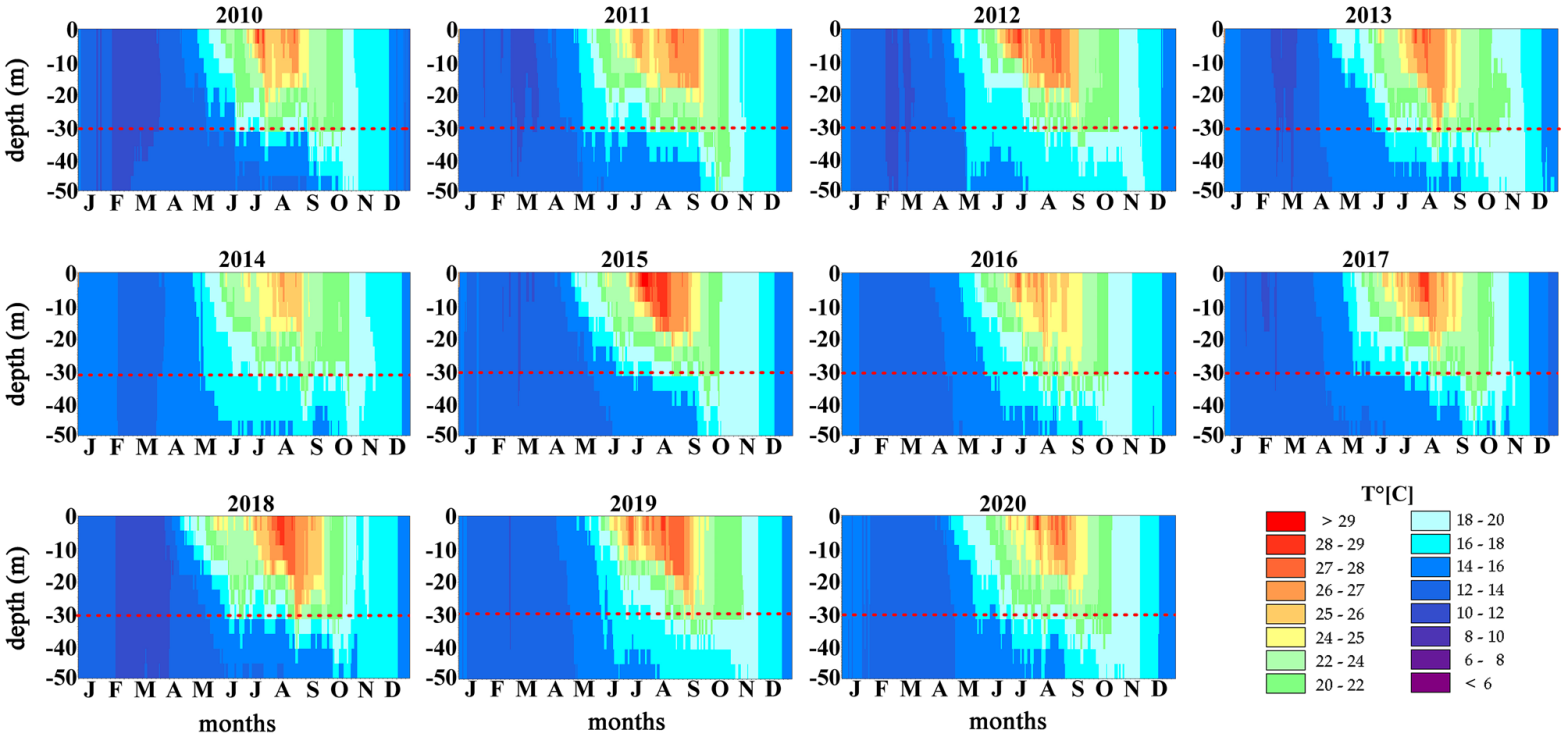
Vertical profiles of water temperature from surface to 50 m depth, from 2010 to 2020, in the Central Adriatic Sea (daily averages from MEDSEA analysis). The dashed red line marks the bathymetry of 30 m.

The observed warming trend (Fig. 6) was in accordance with the vertical profiles of mean water temperature at Tremiti Islands that showed a general increasing in the last 10 years, with a marked warming trend from 2015 up to date (Fig. 7). In particular, daily water-column temperature data from the Mediterranean Sea Physical Analysis and Forecasting product (MEDSEA) showed the increasing of water temperature during late spring and summer, from the surface down to 50 m depth. Seawater temperature below 30 m depth broadly increased of ~ 2 °C from 2016, particularly from late spring to early autumn, matching with the macroalgal overgrowth and the *P. clavata* MME observed. The higher temperatures reached around 30 m depth in 2018 and 2019, compared to previous years, match with the overgrowth of macroalgae/mucilage and the high mortality measured in 2019. In particular, 2018 was the year that reached the highest temperatures at 30 m depth, with 27 °C.

**Figure 8:**
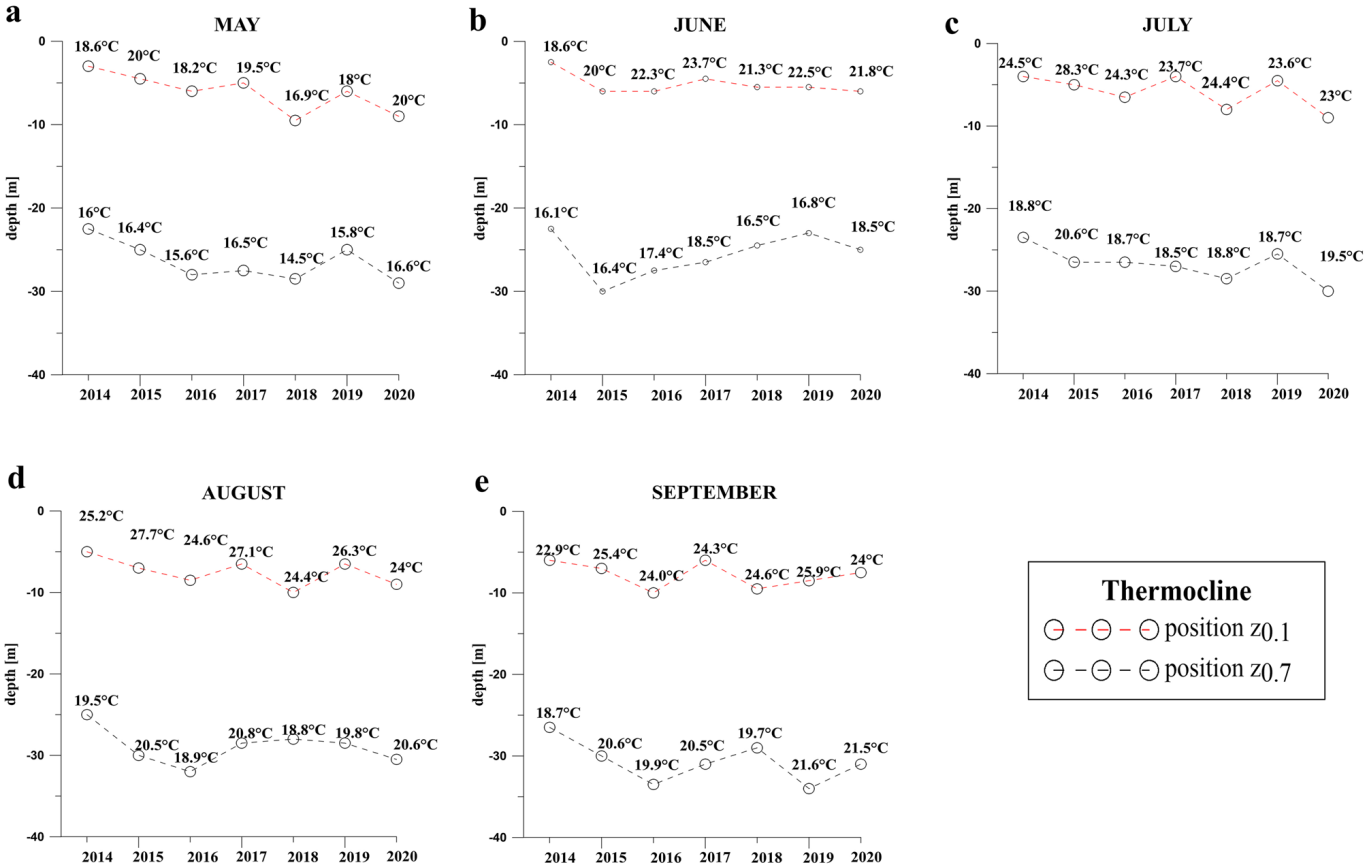
Position of thermocline at Tremiti Islands during late spring and summer, from 2014 to 2020, modelled using MIKE 3 FM HD based on measured data. (**a**) May; (**b**) June; (**c**) July; (**d**) August and (**e**) September.

The 3D hydrodynamic numerical model MIKE 3 FM HD^33^ showed that the lower limit of the thermocline (z_0.7_) was always shallower than 25 m depth until 2015, when it reached 30 m depth in June. Then 2016 resulted critical, with z_0.7_ values dropping down to 33 m depth during August and September. In the following two years, z_0.7_ was slightly shallower than during 2016, but anyway oscillating around 30 m depth (Fig. 8). Finally, z_0.7_ went down to 34 m in September 2019, representing the deepest value observed.

### Additional observations of macroalgal epibiosis on Mediterranean coral forests

**Figure 9:**
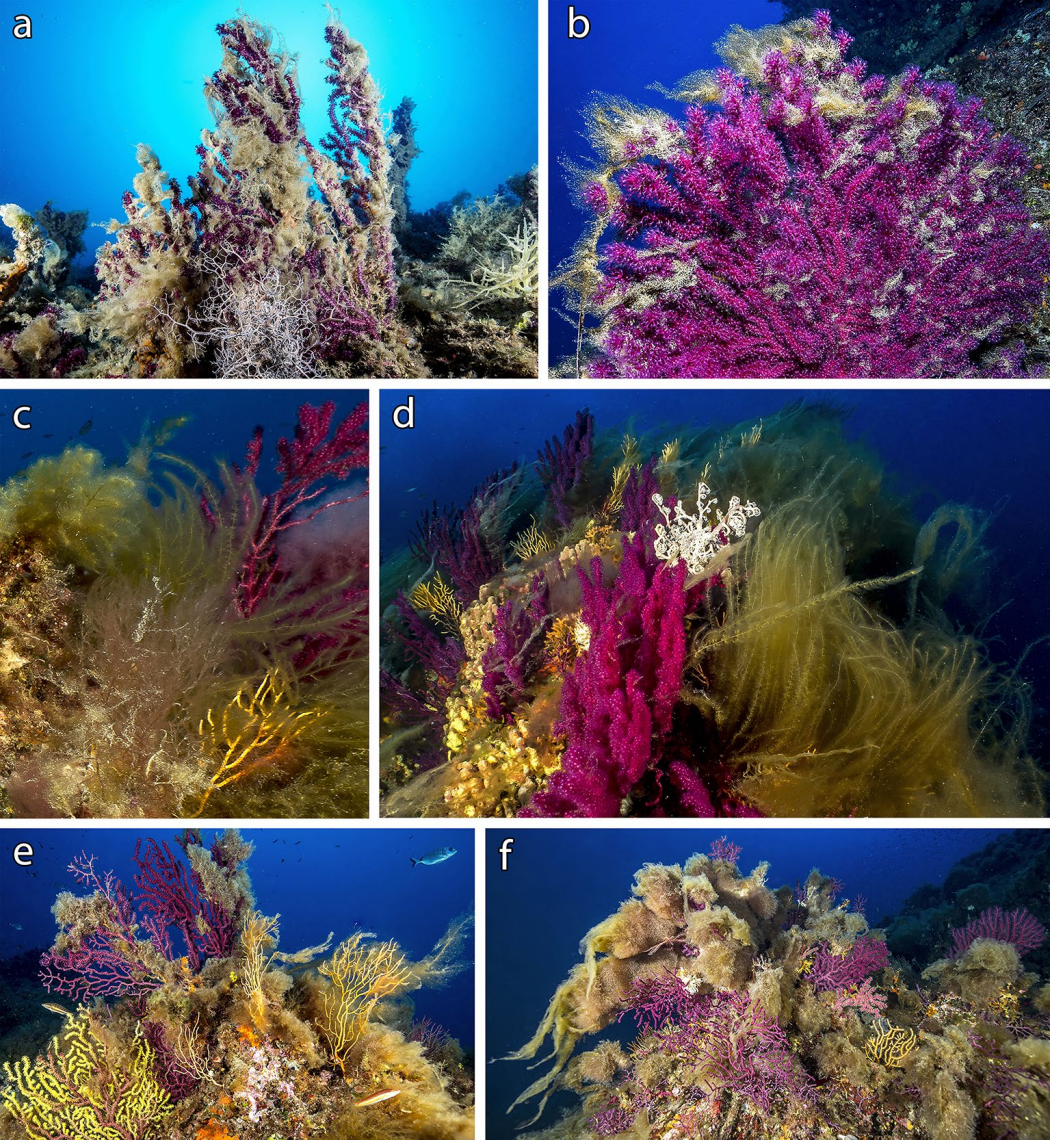
Macroalgae overgrowth and epibiosis on gorgonians. Tuscan Archipelago: (**a**) Off Argentario (35 m depth); (**b**) Off Giannutri (40 m depth); (**c**–**d**) Mezzocanale Shoal (45–50 m depth) with abundant presence of large thalli of *Sporochnus pedunculatus*. Tavolara Marine Protected Area: (**e**–**f**) Papa Shoal (35–40 m depth).

Although not quantified in detail, further records of macroalgal epibiosis on gorgonians occurred in the same period in other Mediterranean areas (Fig. 1). During 2019 and 2020, several macroalgae were observed covering living *P. clavata* and *E. cavolini*, as well as other habitat formers at the Tuscan Archipelago (Tyrrhenian Sea), particularly off Argentario Mount (Fig. 9a), off Giannutri Island (Fig. 9b), off Giglio Island and at Mezzocanale Shoal (Fig. 9c–d). A similar situation was found during 2019 at Papa Shoal, northeast Sardinia, within the Tavolara MPA (Fig. 9e–f).

## Discussion

The loss of habitat complexity caused by extensive MMEs of gorgonians, such as *P. clavata*, is rising a wide concern among scientists because it affects species diversity and alters ecosystem functioning, with the concomitant loss of ecosystem goods and services^18,30^. Global warming and seawater nutrients increasing due to both natural and anthropogenic events are the main triggers of microalgae blooms, including those of mucilage-forming species^34^. In 2017 and 2018, several Mediterranean areas experienced a massive production of mucilage that, once on the seabed, persisted for several months covering the benthic communities. In the case of Tremiti Islands MPA, the mucilage production seemed to be due to a multispecific assemblage of Bacillariophyceae^35^ whose mucus entirely covered the benthic communities. This massive mucilaginous bloom certainly was a major stressor, but not the only one. In fact, with the main part of thermocline moving in deeper layers during summer months, benthic communities normally present below the thermocline were exposed to unusually higher temperatures that, eventually, represented a further source of stress. These disturbance episodes could have compromised the health of *P. clavata* colonies, their capacity to regulate their microbiome through the production of antimicrobial and quorum-sensing interfering compounds^36^ and their resistance toward macroalgae colonization. This represented a combination of factors leading to the MME in a new and more dramatic way. In fact, gorgonians MMEs have been reported thus far consisting in rapid tissue loss and necrosis of the whole colony due to thermal stress (e.g.^17,18,20,25,37–39^), with the following colonization of the dead coral. On the contrary, the colonies we found were still alive when massively colonized by macroalgae. The presence of topical necrotic lesions of the terminal branches (1–3 cm) is a further evidence of the high stress conditions of *P. clavata* and, in many cases, it facilitated the settlement of macroalgae. It is likely that the macroalgal thalli settled on the necrotic extremities also caused a stress by contact to the living polyps nearby, with a positive feedback on the necrosis and the consequent epibiosis. However, pioneer algae such as *S. pedunculatus* and *P. littoralis* were also observed colonizing directly the living branches of *P. clavata*, suggesting a certain weakening of the corals’ defense against epibiosis. Although corals could eventually recover after thermal stress and mucilaginous blooms (causing polyps mortality due to stifling and mechanical impacts, with extremities necrosis)^24^, the macroalgal overgrowth/epibiosis enhances the MME with more devastating effects.

It is noteworthy the unusual invasive behaviour of *S. pedunculatus*, a cosmopolitan species from cold/temperate to tropical waters^40^, but never observed as epibiont of gorgonians. In previous floristic studies carried out at Tremiti Islands^41^, this brown alga was found only in the euphotic zone and reported as very rare, while it is now highly common on gorgonian forests in the upper mesophotic zone. *S. pedunculatus* exhibits a heteromorphic life cycle: macroscopic thalli collected on *P. clavata* were sporophytes, while the microscopic ectocarpoid gametophytes occur in the winter period. This succession of asexual (macro-sporophyte) and sexual (micro-gametophyte) stages could allow the gorgonians recovery during winter, despite the damages caused by the sporophyte can be irreparable, representing a permanent threat. In fact, colonies affected by *S. pedunculatus* became more susceptible to colonization by other epibionts, including alien species. Non-indigenous macroalgae have proved to cause lower survivorship, higher necrosis rates and lower biomass in juvenile *P. clavata* colonies exposed to algal overgrowth^42^.

The co-occurring *P. littoralis* is a fast-growing brown alga with a wide distribution in cold and temperate zones of both hemispheres^40^, considered introduced and well established in the Mediterranean basin^43^. However, this is the first record of *P. littoralis* in the study area^41^. It is usually well developed during spring and early summer, starting to detach and decay in mid-summer. This species, like other Ectocarpales, produces polysaccharides and humic acids involved in mucilage production^44^, representing a potential contributor in the mucilaginous aggregates. The negative effects of *P. littoralis* have been already observed, for instance, on fish egg survival and on the recruitment of the kelp *Fucus vesiculosus* Linnaeus^45^.

Macroalgae overgrowth as epibiont on living corals simultaneously represent a consequence and a cause of the corals’ stress. Mechanism by which macroalgae impair the gorgonians could be various, from the physical effects of overgrowth and tissue necrosis to allelopathic interactions, and are likely to occur in already stressed populations. In fact, colonies on vertical substrate were less affected by mucilaginous aggregates and resulted overall healthier than those on horizontal and sub-vertical substrates, as well as less affected by macroalgal epibionts. Their deeper occurrence and their position on a vertical substrate can reduce the entity of the stress because of colder water, a reduced light availability for algal growth and a minor trapping of mucilaginous masses that are stopped by the shallower gorgonians. Moreover, colonies on vertical substrate are generally less affected by mucilage persistence because of a high exposure to currents that remove mucilaginous aggregations.

Algae overgrowth and epibiosis matched with the increasing of SST (enhanced by heat waves), the consequent warming of the water at lower layers and the lowering of the thermocline that naturally protects the upper mesophotic communities during summer. The ongoing algal epibiosis on *P. clavata* seems to be widespread in the study area and at Mediterranean level. It affects also other gorgonian species, such as those of the genus *Eunicella*, and probably other habitat formers of the mesophotic zone. Besides the presence of extreme values, this warming trend resulted related to a homogeneous global increase of high SST records in the area, as also observed at basin scale^32^.

Both empiric observations and future projections (e.g.^46,47^) show the increase in Mediterranean SST, with more frequent occurrence of extreme ocean warming events. The effects on coral forests and their biodiversity include high level of coral mortality and low or null recruitment. Deep areas can represent a refuge against global warming, but the current trend could lead to the disappearance of species such as *P. clavata* from the upper mesophotic zone, particularly if coupled with other anthropogenic impacts (e.g. ^29,30,37,38,42,48,49^). This can have a negative effect on the ecology and the functioning of such ecotone area between the euphotic and the mesophotic zones, as well as negative effects on human activities including tourism and recreation. In fact, *P. clavata* forests display a high aesthetic value, representing the main attraction for scuba diving tourism in many areas^49–51^. Punta Secca shoal is considered one of the most appreciated diving sites in the Adriatic Sea, representing an important source of income for the local community that is currently switching from a fishery-based tourism toward more sustainable uses also thanks to the presence of a MPA^50^.

## Conclusions

Global warming and the increasing frequency of heat waves are stressing *P. clavata* and other important habitat formers of the mesophotic zone. Their time for recovery is further reduced by the consequent occurrence of mucilaginous blooms and macroalgae overgrowth/epibiosis on stressed corals. This is producing a cascade effect that could be irreversible, with lush and diversified benthic communities shifting to monotonous ones.

Short-time temperature anomalies, warming trend and thermocline lowering are compromising centuries of coral communities within few years. Despite protected against fishing pressures thanks to MPAs, coral forests are undergoing MMEs, showing that local habitat protection affords little or no resistance to global warming. These effects are now evident, highlighting once more the urgency of global actions to curb future warming.

## Materials and methods

### Study area

The study is focused on Tremiti Islands MPA (Adriatic Sea), located 12 nautical miles north of the Gargano promontory (Apulia Region, Southern Italy) (Fig. 1). The MPA involves an archipelago which consists of five islands with a gradient of restrictions^9^. We monitored in detail Punta Secca Shoal, at the northeast extremity of Caprara Island (Table S2), that represents one of the most appreciated diving site of the area due to its lush population of *P. clavata*^50^. This area is included in the highly protected zone of the MPA (Zone B), where anchoring and recreational fishing are forbidden. The top of the shoal is located at 6 m depth and is characterized by photophilous algae on rocky bottom. The seabed falls quite rapidly, alternating slumped blocks and vertical cliffs. At 32–35 m depth a wide, almost-flat rocky area is characterized by coralligenous bioconstructions (*sensu*^52^). An extensive forest of *P. clavata* starts here and continues along the slope of the shoal (35–38 m depth) and on part of the following vertical wall, up to 70 m depth. This area is characterized by a complex morphology, enhanced by the bioconstruction activity of typical coralligenous calcifying organisms (e.g. calcareous red algae, serpulids, bryozoans, corals) as well as patchy aggregations of the oyster *Neopycnodonte cochlear* (Poli, 1795).

Seven different sites characterized by forests of *P. clavata*, *E. cavolini* and/or *A. subpinnata* were selected around the archipelago (35–80 m depth) to monitor the status of these vulnerable marine ecosystems (Fig. 1; Table S2). Coral forests characterized by *P. clavata* and *Eunicella* spp. were also surveyed in the Tyrrhenian Sea, in order to have a preliminary record of mucilaginous aggregations and macroalgal epibiosis from the Western Mediterranean Sea. In particular, five sites were surveyed at the Tuscan Archipelago and one at Tavolara MPA (Fig. 1; Table S2). All these sites were qualitatively monitored to identify coral mortality, mucilaginous blooms and macroalgal epibiosis.

### Video recording and analyses

The population of *P. clavata* at Punta Secca shoal was monitored by scuba diving during the summer of 2014–2019. Technical dives were carried out in three different portions of the shoal characterized by the coral forest. In particular, the horizontal seabed at 32–35 m depth, the sub-vertical slope at 35–38 m depth and the upper part of the vertical wall at 38–41 m depth were surveyed. Video transects of 50 × 2 m were carried out within the *P. clavata* forest at each area (three replicates per area), using a 4 K video camera equipped with a light apparatus of 10,000 lm and two parallel laser beams for scaling. Videos were analysed using Adobe Premiere Pro software. Sampling units of 2.5 ± 0.2 m^2^ were defined along each transect, according to the minimal area used for mesophotic benthic communities in the Mediterranean Sea^8,53–55^. Density (living colonies m^−2^) and mortality (dead colonies m^−2^) of *P. clavata* were calculated for each sampling unit and expressed as mean ± standard error. Transects were georeferenced using a transponder mounted on the divers and a differential GPS with an accuracy of 0.1 m. Thanks to the precise positioning and identifiable reference points on the seabed, it was possible to return exactly on the same transect during every year of monitoring and compare the living corals’ density.

Statistical analysis were performed using PAST 4.03. A test for equal means was used to test the homogeneity of the three replicates within each of the three substrates in both 2014 and 2019. Data distribution for each transect was not normal (Shapiro–Wilk W test, *p* > 0.05), so differences among the coral density in 2014 and 2019, within the same substrate, were tested using a Kruskal–Wallis test for equal medians.

Colonies affected by epibiosis were also quantified as colonies m^−2^ and distinguished by three main macro-categories of epibionts: macroalgae, mucilage and others (e.g. invertebrates and eggs).

### Algal epibionts

Qualitative and quantitative analyses were performed on the necrotic apical fragments of living gorgonians and on the algal skeins densely covering the coral colonies. Samples of gorgonian distal branches (portions 10 cm long with necrotic extremities) and algal skeins were randomly collected during September 2019 from living colonies on both horizontal and sub-vertical hard bottoms.

Basal portions of erect thalli, encrusting or mat-forming algae were removed from the apical fragments of gorgonians by scraping with a chisel. Fresh weight of each species occurring both on the terminal branches and in the skeins was expressed as percentage.

Fresh material was observed under a Leica MZ 7.5 stereomicroscope (Leica, Wetzlar, Germany). For morphological observations, squash preparations and sections of thalli, obtained by free-hand cutting or with a DSK-1000 vibratome (Dosaka, Kyoto, Japan), were opportunely stained. Photomicrographs and measurements were made using an Olympus BX-40 light microscope (Olympus, Melville, USA) fitted with an Olympus DP21 digital camera (Olympus, Melville, USA).

Non-indigenous species (*sensu*^43^) were highlighted. The nomenclature of the identified taxa followed AlgaeBase^40^.

### Satellite measurements of sea surface temperature

Sea surface temperature (SST) database extracted from the MODerate-resolution Imaging Spectroradiometer (MODIS) on board the NASA Aqua satellite has been collected from the NASA archive (https://oceancolor.gsfc.nasa.gov/cgi/browse.pl) and used to analyse the study area with a spatial resolution of 1 × 1 km. SST was extracted from the Thermal Infrared (IR) bands data, i.e., the bands 31 and 32 (λ = 11 and 12 μm, respectively). The functional form used to derive SST from MODIS data is based on a modified version of the nonlinear SST algorithm (NLSST)^56^ and uses empirical coefficients derived by regression of collocated in situ and satellite measurements^57^.

The collected dataset consisted in daily SST products from July 2002 to September 2020. The measures distributed over a window of 0.3° latitude × 0.3° longitude around Tremiti Islands were spatially averaged. Data were aggregated to obtain a monthly time series, then the seasonal-trend decomposition based on LOcally wEighted Scatterplot Smoothing (LOESS), known as STL^32,58^, was applied using R software (STL implemented in stats-package). This filtering procedure was used to identify the trend and the residual components from the seasonal times series.

### Hydrodynamic model and vertical profiles of water temperature

The 3D hydrodynamic numerical model MIKE 3 FM HD produced by the Danish Hydraulic Institute^33^ was performed to describe the water temperature patterns up to 50 m depth in the study area, from 2014 to 2018. The model is based on the numerical solution of the three-dimensional incompressible Reynolds averaged Navier–Stokes equations subject to the assumptions of Boussinesq and of hydrostatic pressure. Thus, the model consists of continuity, momentum, temperature, salinity and density equations, with a turbulent closure scheme. The density does not depend on the pressure, but only on the temperature and the salinity.

The hydrodynamic simulation for 7 years (2014–2020) was carried out in a baroclinic model in order to improve the numerical approach and model more realistic conditions. Temperature and salinity vertical profiles were extracted by the Mediterranean Sea Physics Reanalysis model, characterized by a horizontal grid resolution of 1/24° (ca. 4.6 km in latitude) and by 72 unevenly spaced vertical levels^59^ (https://marine.copernicus.eu). The atmospheric data (u and v components of wind, atmosphere pressure, total cloud cover, solar radiation and air temperature), available every 6 h, were extracted using ERA-Interim developed through the Copernicus Climate Change Service^60^. The precipitation data, available every 1-day, was predicted by CPC Merged Analysis of Precipitation (CMAP)^61^. The turbulent closure model used within the MIKE 3 FM HD model relies on the k-ε formulation for the vertical direction^62^ and on the Smagorinsky formulation for the horizontal direction^63^. The Smagorinsky coefficient has been assumed uniform in space and temporally constant, equal to 0.6. Simulations were performed by adopting a seabed roughness equal to 0.1 m, according to recent sensitivity analysis^64^, and a wind drag coefficient C_d_ equal to 0.002, based on earlier studies (e.g.^9,64–66^).

The model was calibrated and validated using data from CTD casts carried out monthly in two sites of the study area. Once verified the validity of the hydrodynamic model, the procedure by^67^ was adopted, considering the variation of temperature across the isothermal layer, thermocline and deep layer as:

1 $$T_{d} = T\left( {z_{1} } \right) - T\left( {z_{b} } \right)$$

where *z*_1_ is the depth of upper layer and *z*_b_ is the depth of the deeper layer.

The main part of the thermocline was identified between the depth *z*_0.1_ and depth *z*_0.7_ with 10% and 70% temperature difference to depth *z*_1_, respectively^67^:

2 $$T\left( {z_{1} } \right) - T\left( {z_{0.1} } \right) = 0.1T_{d}$$

3 $$T\left( {z_{1} } \right) - T\left( {z_{0.7} } \right) = 0.7T_{d}$$

Finally, daily water column temperature at 12:00 UTC was downloaded from the European Union Copernicus Marine Environment Monitoring Service in order to analyse the temporal trend of water temperature in the Central Adriatic Sea. Water temperatures for years 2010–2020 were obtained from the Mediterranean Sea Physical Analysis and Forecasting product (MEDSEA), characterized by a horizontal grid resolution of 1/24° (ca. 4.6 km) that included Tremiti Archipelago, and by 141 unevenly spaced vertical levels^68^.

## Supplementary Information

Supplementary Tables.

## Data Availability

The datasets generated during and/or analysed for the current study are available from the corresponding author upon request.
