# Supplementary material for: Effects of global warming on Mediterranean coral forests
Source: Sci Rep. 2021 Oct 19;11:20703. doi: 10.1038/s41598-021-00162-4 (PMC8526741; doi:10.1038/s41598-021-00162-4)
Supplement: Supplementary file 1 — Supplementary Tables. [file 41598_2021_162_MOESM1_ESM.docx]

**Effects of global warming on Mediterranean coral forests**

Giovanni Chimienti^1,2*^, Diana De Padova^2,3^, Maria Adamo^4^, Michele Mossa^2,3^, Antonella Bottalico^1^, Anna Lisco^1^, Nicola Ungaro^5^, Francesco Mastrototaro^1,2^

^1^Department of Biology, University of Bari Aldo Moro, Bari, Italy

^2^CoNISMa, Roma, Italy

^3^Polytechnic University of Bari, DICATECh, Bari, Italy

^4^Institute of Atmospheric Pollution Research (IIA), National Research Council (CNR), Bari, Italy

^5^Apulian Regional Agency for the Environmental Prevention and Protection, Bari, Italy

*Correspondence: G. Chimienti, Department of Biology and CoNISMa LRU, University of Bari Aldo Moro, Via Orabona 4, 70125 Bari, Italy. Tel: +39 080 5443330. E-mail: giovanni.chimienti@uniba.it

| **Shoal area** | | | **Top** | **Slope** | **Flank** |
| --- | --- | --- | --- | --- | --- |
| **Depth (m)** | | | 32–35 | 35–38 | 38–41 |
| **Inclination** | | | horizontal | sub-vertical | vertical |
| **Desnity (colonies m^-2^)** | | 2014 | 10.92 ± 0.18 | 11.54 ± 0.29 | 12.48 ± 0.15 |
|  |  | 2019 | 6.62 ± 0.18 | 6.09 ± 0.15 | 11.24 ± 0.17 |
| **Mortality (dead colonies m^-2^)** | | 2014 | 0.34 ± 0.03 | 0.26 ± 0.04 | 0.33 ± 0.03 |
|  |  | 2019 | 5.61 ± 0.13 | 5.32 ± 0.16 | 0.58 ± 0.02 |
| **Epibiosis** | No epibionts | 2014 | 10.68 ± 0.19 | 10.91 ± 0.28 | 11.83 ± 0.14 |
|  |  | 2019 | 0.22 ± 0.03 | 0.27 ± 0.05 | 8.08 ± 0.10 |
|  | Mucilage | 2014 | 0.18 ± 0.02 | 0.08 ± 0.02 | 0.31 ± 0.03 |
|  |  | 2019 | 0.00 ± 0.00 | 0.00 ± 0.00 | 0.00 ± 0.00 |
|  | Other | 2014 | 0.06 ± 0.01 | 0.55 ± 0.05 | 0.64 ± 0.03 |
|  |  | 2019 | 0.07 ± 0.02 | 0.00 ± 0.00 | 0.08 ± 0.01 |
|  | Macroalgae | 2014 | 0.00 ± 0.00 | 0.00 ± 0.00 | 0.00 ± 0.00 |
|  |  | 2019 | 6.32 ± 0.18 | 5.82 ± 0.15 | 3.07 ± 0.12 |

**Table S1.** Density and mortality of *Paramuricea clavata* during 2014 and 2019 on three different substrates. Type and quantification of the epibiosis is also provided. Data are expressed as mean ± standard error.

| **Site** | **Depth (m)** | **Latitude** | **Longitude** | **Habitat** |
| --- | --- | --- | --- | --- |
| **Tuscan Archipelago, Tyrrhenian Sea** | | | | Isolated colonies of *P. clavata.* |
| Off Argentario Mount | 30–40 | 42° 24.064’ N | 11° 05.474’ E | Forest of *P. clavata.* |
| Giannutri Island, Punta Scaletta | 30–40 | 42° 15.471’ N | 11° 05.661’ E | Forest of *P. clavata* and *E. cavolini* on both horizontal and vertical substrates. |
| Giannutri Island, Punta Secca | 30–40 | 42° 15.848’ N | 11° 06.559’ E | Forest of *P. clavata* on both horizontal and vertical substrates, with isolated colonies of *E. cavolini*. |
| Giglio Island, Le Scole | 25–35 | 42° 21.361’ N | 10° 55.906’ E | Forest of *P. clavata* and *E. cavolini* on both horizontal and vertical substrates. |
| Mezzocanale Shoal | 25–50 | 42° 20.403’ N | 11° 05.519’ E | Forest of *P. clavata* and *E. cavolini* on both horizontal and vertical substrates. |
| **Tavolara Island, Tyrrhenian Sea** | | | |  |
| Papa Shoal | 35–45 | 40° 54.743’ N | 09° 44.678’ E | Forest of *P. clavata* and *E. cavolini.* |
| **Tremiti Archipelago, Adriatic Sea** | | | |  |
| Caprara, Punta Secca Shoal | 30–70 | 42° 08.435’ N | 15° 31.533’ E | Forest of *P. clavata* on both horizontal and vertical substrates. Isolated colonies of *E. cavolini*. |
| Caprara, Parete nera | 30–80 | 42° 08.376’ N | 15° 31.752’ E | Forest of *P. clavata* and *A. subpinnata* on both horizontal and vertical substrates. |
| Caprara, Architiello | 30–80 | 42° 08.332’ N | 15° 30.376’ E | Forest of *P. clavata* with *E. cavolini* and, in the deepest part, mixed forest with *A. subpinnata*. |
| San Nicola, Punta del Cimitero | 60–65 | 42° 07.488’ N | 15° 31.322’ E | Occasional *P. clavata*, *E. cavolini* and forest of *A. subpinnata*. |
| San Nicola, Est | 35–45 | 42° 07.278’ N | 15° 30.758’ E | Isolated colonies of *E. cavolini*. |
| San Domino, Punto 55 | 35–50 | 42° 06.578’ N | 15° 29.866’ E | Forest of *P. clavata* and *E. cavolini* on both horizontal and vertical substrates. |
| San Domino, Punta Zio Cesare | 45–70 | 42° 05.881’ N | 15° 28.852’ E | Forest of *E. cavolini*. |
| San Domino, Faro | 35–55 | 42° 06.273’ N | 15° 28.422’ E | Forest of *P. clavata*. |

**Table S2.** Surveyed depth, geographic coordinates and habitat type at the study sites, with indication of the presence of *Paramuricea clavata*, *Eunicella cavolini* and *Antipathella subpinnata*.
